# Supplementary figures and images for: Onion pollenkitt: Function, DNase activity, fatty acid composition, and SEM imaging
Source: PLoS One. 2025 Apr 7;20(4):e0321197. doi: 10.1371/journal.pone.0321197 (PMC11975101; doi:10.1371/journal.pone.0321197)

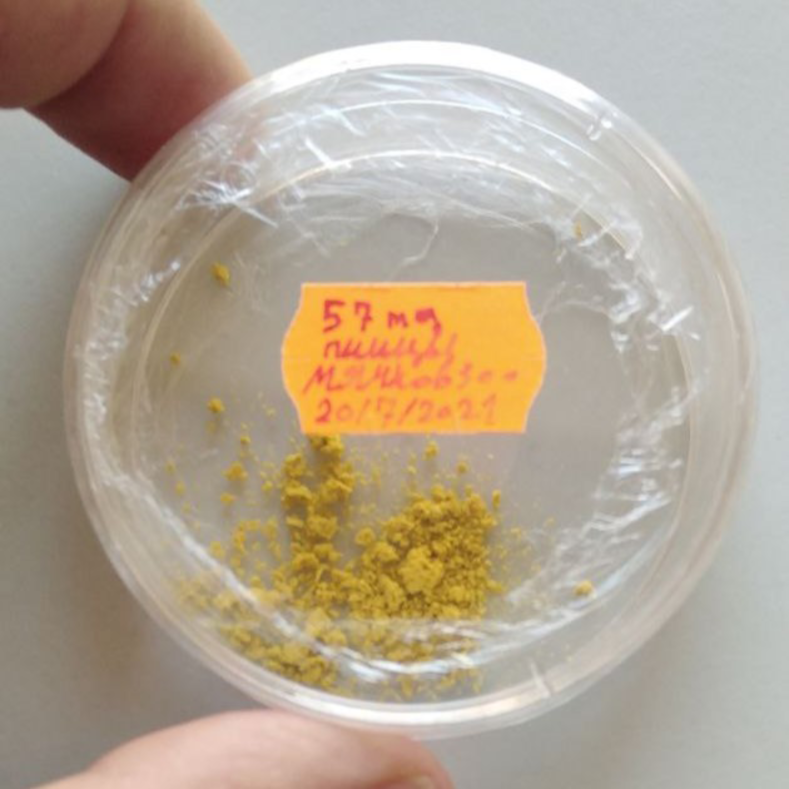

Supplement: S1 Fig — (TIF) [file pone.0321197.s001.tif]

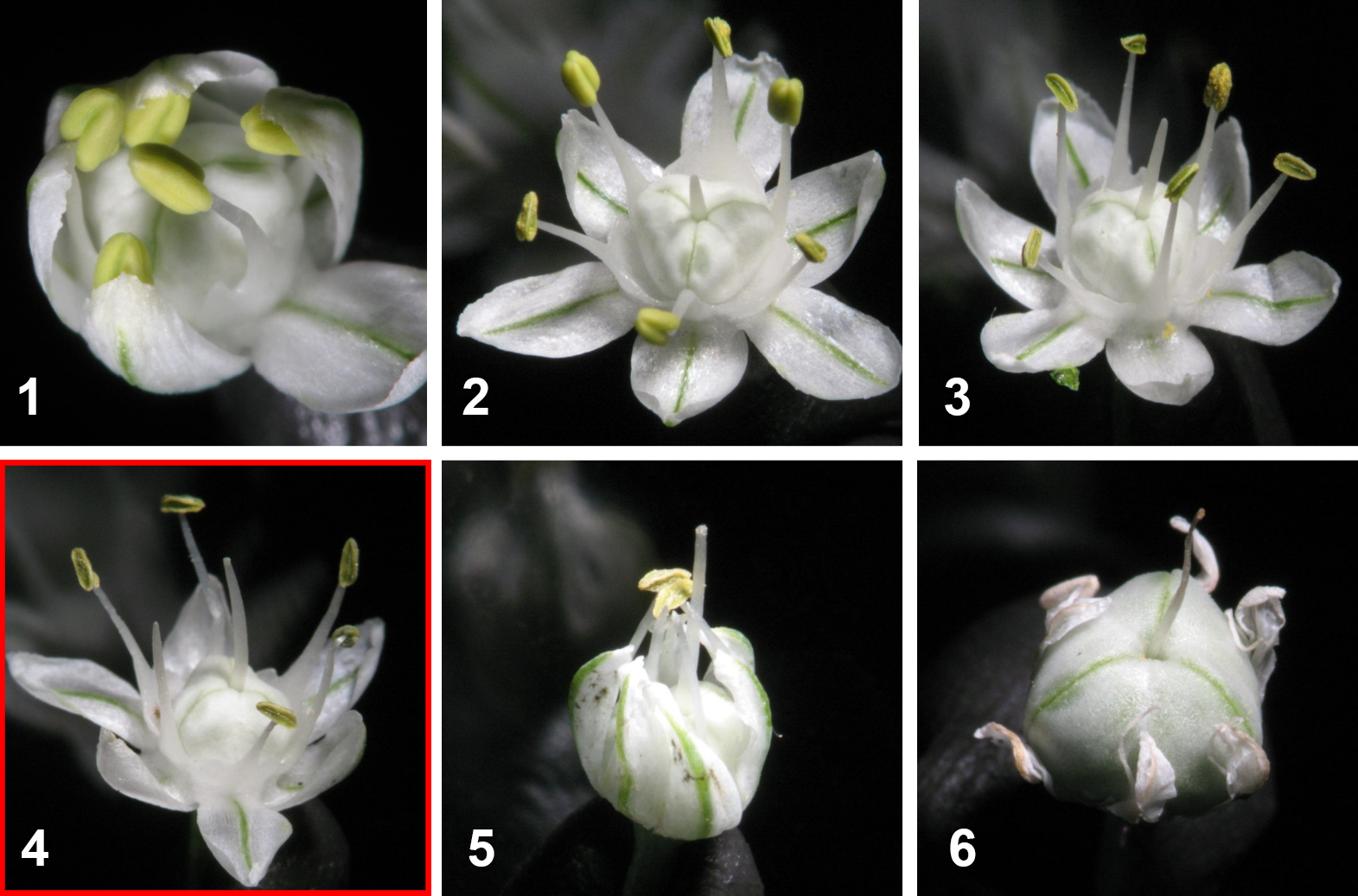

Supplement: S2 Fig — In our study, pollination was performed during the appearance of the stigmatic knob (stage 4). (TIF) [file pone.0321197.s002.tif]

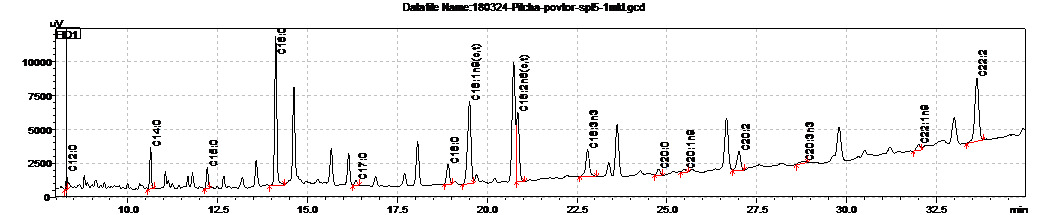

Supplement: S3 Fig — chromatogram showing the separation of fatty acid methyl esters The x-axis represents retention time in minutes, while the y-axis indicates the detector response in microvolts (uV). (TIF) [file pone.0321197.s003.tif]
