# Supplementary material for: Onion pollenkitt: Function, DNase activity, fatty acid composition, and SEM imaging
Source: PLoS One. 2025 Apr 7;20(4):e0321197. doi: 10.1371/journal.pone.0321197 (PMC11975101; doi:10.1371/journal.pone.0321197)
Supplement: S1 Table — (PDF) [file pone.0321197.s004.pdf]

S1 Table. The results of GC-FID of onion pollenkitt: retention time (minutes) and peak area (arbitrary unit).

**Fatty acid**		**Retention time (minutes)**	**Area**
12:0	Lauric	8.33	4590
13:0	Tridecylic	9.4	3052
14:0	Myristic	10.7	15584
9-14:1	Myristoleic	11.3	3603
15:0	Pentadecylic	12.3	11343
16:0	Palmitic	14.22	64623
17:0	Margaric	16.48	4964
10-17:1	10-heptadecaenoic	17.03	18425
18:0	Stearic	19.05	13580
9-18:1	Oleic	19.66	56866
9,12-18:2	Linoleic	21.01	41204
9,12,15-18:3	α-linolenic	22.97	17431
20:0	Arachidic	24.94	4439
9-20:1	cis-11-Eicosenoic	25.67	2936
11,14-20:2	Eicosadienoic	27.20	14241
8,11,14-20:3	cis-8,11,14-Eicosatrienoic	28.14	1659
5,8,11,14-20:4	Arachidonic	29.65	4560
22:0	Behenic	31.41	4049
9-22:1	Erucic	32.21	5630
13,16-22:2	Docosadienoic	33.84	52334
