# Supplementary material for: Onion pollenkitt: Function, DNase activity, fatty acid composition, and SEM imaging
Source: PLoS One. 2025 Apr 7;20(4):e0321197. doi: 10.1371/journal.pone.0321197 (PMC11975101; doi:10.1371/journal.pone.0321197)
Supplement: Attachment — Submitted filename: Response to Reviewers.docx [file pone.0321197.s005.docx]

**Response to Reviewer #1:**

This paper provides a comprehensive examination of onion (*Allium cepa*) pollenkitt, focusing on its functional properties, biochemical composition, and structural features. The study is intriguing as it delves into an often-overlooked aspect of plant reproductive biology while employing a combination of biochemical assays and imaging techniques.

Our response:

Thank you for your careful reading of our manuscript and deep understanding of our idea motivating the study. We appreciate your positive evaluation.

**Response to Reviewer #2**

**General comment:**

“Manuscript is dealing with interesting topic. Results are fairly presented. I suggest that impersonal style should be used through the text. However, some issues need to be answered: English must be checked. Typographical errors occur through the text. All abbreviations used in the text must be explained when first mentioned. Latin names of plants must be written in Italic.

In general discussion needs to be improved. References should be prepared by the journal instructions. For the figures: all abbreviations should be explained in the table legend”

Our response:

Thank you for your thoughtful comments and the time you spent reviewing our manuscripts. Regarding your suggestions, we have made the following revisions:

- The manuscript has been checked for English language and typographical errors, and corrections have been made throughout. We also removed all unnecessary hyphenations.
- All abbreviations are fully explained at the first mention.
- The Latin names of plants have been italicized.
- Necessary changes have been made to the references format.
- All abbreviations in the figure legends have been explained as suggested.

We appreciate your valuable comments and believe these changes have improved the manuscript. Below is a step-by-step response to each of the specific comments you provided.

**Specific comments:**

| **Reviewer’s remark** | **Our response** |
| --- | --- |
| “Lines: 65-66: … fatty acids could be classified as short-chain (1-5 carbon atoms). Please correct it, as fatty acids are those with C4 and more carbon atoms.” | Thank you for your comment. We agreed with you. We have analyzed the literature on this issue. There is no generally accepted classification of fatty acids. This part of the Introduction has been removed and accordingly the following text has been added to the Discussion: “Most naturally occurring fatty acids have an unbranched chain with 4 to 28 carbon atoms” (lines 371-372) |
| “Lines 72-79 should be deleted. Comparison to other topics is redundant as this is not a review paper.” | We find it valuable to include this paragraph in the manuscript, particularly in the introduction. By highlighting the scarcity of research on pollenkitt, particularly on agriculturally important species, we draw attention to the need for further research in this area. |
| “Lines 92-97: Text starting with: Here, we further explore… should be placed in a separate paragraph as it underlines the aim of the work.” | Thank you for pointing this out. We have revised the manuscript accordingly. |
| “Line 172: dH2O …. Correct it to dH2O. What is d?” | Thank you for your comment. The "d" in dH2O stands for "distilled". We have added an explanation of this abbreviation in the text where it first appears (line 162). Additionally, the "2" in H_2_O has been correctly formatted as a subscript throughout the manuscript |
| “Line 172: …ddH2O…. Correct it to ddH2O. What is dd?” | This is a typo. It should be dH_2_O. Thank you for pointing this out. |
| “Line 201: …the upper hexane layer containing methyl esters. ..methyl esters of what? Be more precise.” | Thank you for pointing this out. We have specified "fatty acid methyl esters". We have made this correction in the text (line 199) |
| “Line 215: Jiapeng Chen and Hongbing Liu [32]. Correct to… Chen and Liu [32].” | Changes have been made to the text accordingly (line 213) |
| Line 217: In the section SEM analysis of onion pollen grains with and without pollenkitt, authors mentioned Fig. 3 a-2. Later in Results (lines 239,240, 243…) authors mentioned Fig 1a-c. And later, in lines 264-270 they mentioned Fig 2a-d, and in line 278 Fig 2f. This should be corrected. Authors should label figures in order as they appear in the text. | Thank you for your comment. We have revised the figure references to ensure they are labeled in the proper order as they appear in the text. |
| “Line 302: * These numbers.. What the asterisk refers to?” | These lines are part of the footnote for Table 1. The asterisk refers to the title of the last column (% in pollenkitt*) |
| “Lines 377: short-chain fatty acids (1-5 carbon atoms). See previous comment about classification of fatty acids” | Changes has been made. |
| “Line 408: what is VLCFAs? Explain.” | VLCFAs is an abbreviation for very long-chain fatty acids. This has already been explained where it first appears in the text (line 410). |

**Response for Reviewer #3**

General comment from the reviewer:

“This work represents a major contribution to plant biology and presents new insights into the role and chemical composition of pollenkitt in onion pollen. A holistic approach combining chemical analysis (GC-FID) with structural and functional investigations (SEM) provides deeper insights into the function of pollenkitt. Several sections of the manuscript need clarification, amplification, or correction in order to enhance the overall quality of the manuscript.”

Our response:

Thank you for the time and effort spent revising our manuscript. We have carefully reviewed all suggestions and made the necessary revisions to improve the manuscript's quality and clarity. We have addressed each of the specific comments in the table below.

Specific comments:

| **Reviewer’s remark** | **Our response** |
| --- | --- |
| “The abstract is too long and does not summarize the main points effectively. Suggestion: The abstract should be shortened while ensuring that the main findings are clearly communicated. Be concise and precise.” | We believe the abstract properly reflects our findings while being concise. Following PLOS ONE's guidelines, which suggest a 300-word limit, our abstract is 175 words. |
| “Introduction: The introduction is good, but it would be even better if it contained a more thorough literature review, especially regarding the physiological functions of pollenkitt, other than just comparing the results of the keyword search.  The suggestion would be the development of sections by incorporating references that illustrate recent research into the biochemical nature and ecological roles of pollenkitt in a variety of plant species.” | The physiological functions of pollenkitt are thoroughly covered in lines 49-57 of the introduction, with sufficient supporting literature (references [6-11]). We believe this section provides a comprehensive overview of the topic in the context of our study. |
| “Results are presented regularly; however, the format of some figures and tables could be improved for better clarity.  Recommendation: Ensure that all figures and tables have concise captions that describe the data presented, and that they are integrated smoothly into the text.” | We reviewed the entire manuscript and made the necessary changes and corrections in the journal’s format to ensure clarity. |
| “Some figure legends are not descriptive enough and lack the explanation of abbreviations.  Suggestion: Legends should be revised to include a brief statement of the significance of each image. Also, all abbreviations should be written out in full the first time they are used.” | We have formatted all figure captions according to the PLOS ONE guidelines. Each caption should be consisted of two main parts: the figure title and the legend. We have ensured that all captions in our manuscript are laid out accordingly. There is no requirement for a "statement of the significance" of the image in the caption (please refer to this link:  https://journals.plos.org/plosone/s/file?id=wjVg/PLOSOne_formatting_sample_main_body.pdf")  Nonetheless, all scientific aspects of the figures are thoroughly presented either in the Results or the Discussion sections.  Regarding the abbreviations, we have reviewed the manuscript and ensured that all abbreviations are written out in full the first time they are used for clarity. |
| “Some methodological steps lack in detail, mainly those related to fatty acids extraction and analysis and DNase activity.  Exhuming: Include more specific protocols, pointing out exactly in which ways they deviated from the standard practice if the protocol was modified; that could be helpful for people in trying to replicate this study further.” | We believe that all technical procedures are presented in sufficient detail to ensure reproducibility. Please refer to the Materials and Methods section, particularly lines 163-192 (on DNase activity analysis) and lines 196-216 (on fatty acid extraction). Additionally, a comprehensive protocol for the SEM experiments is thoroughly presented in our earlier paper (Ermolaev et al., 2024), which is also properly cited in the manuscript [37]. |
| “While the discussion well summarized the findings, its practical implications of DNase activities, especially in relation to genetic engineering, can further be elaborated on.  Suggestion: Add an explanation of the consequences that these results have when attempting applications, specifically enhancements on PMGE.” | This issue is clearly addressed in many places of the manuscript (lines 336-354) and also in the Conclusion section. |
| “The conclusion does summarize the research, but could be stronger  Suggestion: Highlight the significance of the pollenkitt not only for onions but also its broader applicability in plant biology and gene editing applications.” | See the previous comment. |
| “There is some inconsistency in the formatting of the references.  Suggestion: Check the format of the citations according to the journal instructions and fix any inconsistencies in the author lists, year of publication or” | Thank you for bringing this to our attention. We have made all necessary changes to the manuscript accordingly. |
